# Treatment of hypophosphatemia in the intensive care unit: a review

**DOI:** 10.1186/cc9215

**Published:** 2010-08-03

**Authors:** Daniël A Geerse, Alexander J Bindels, Michael A Kuiper, Arnout N Roos, Peter E Spronk, Marcus J Schultz

**Affiliations:** 1 Department of Intensive Care Medicine, Catharina Hospital Eindhoven, Michelangelolaan 2, Eindhoven, 5623 EJ, The Netherlands; 2 Department of Intensive Care Medicine, Medical Centre Leeuwarden, PO Box 888, Leeuwarden, 8901 BR, The Netherlands; 3 Department of Intensive Care Medicine, Academic Medical Center, University of Amsterdam, Meibergdreef 9, Amsterdam, 1105 AZ, The Netherlands; 4 HERMES Critical Care Group, Meibergdreef 9, Amsterdam, Amsterdam, 1105 AZ, The Netherlands; 5 Department of Intensive Care Medicine, Gelre Hospitals, location Lukas, PO Box 9014, Apeldoorn, 7300 DS, The Netherlands; 6 Laboratory of Experimental Intensive Care and Anesthesiology (LEICA), Academic Medical Center, University of Amsterdam, Meibergdreef 9, Amsterdam, 1105 AZ, The Netherlands

## Abstract

**Introduction:**

Currently no evidence-based guideline exists for the approach to hypophosphatemia in critically ill patients.

**Methods:**

We performed a narrative review of the medical literature to identify the incidence, symptoms, and treatment of hypophosphatemia in critically ill patients. Specifically, we searched for answers to the questions whether correction of hypophosphatemia is associated with improved outcome, and whether a certain treatment strategy is superior.

**Results:**

Incidence: hypophosphatemia is frequently encountered in the intensive care unit; and critically ill patients are at increased risk for developing hypophosphatemia due to the presence of multiple causal factors. Symptoms: hypophosphatemia may lead to a multitude of symptoms, including cardiac and respiratory failure. Treatment: hypophosphatemia is generally corrected when it is symptomatic or severe. However, although multiple studies confirm the efficacy and safety of intravenous phosphate administration, it remains uncertain when and how to correct hypophosphatemia. Outcome: in some studies, hypophosphatemia was associated with higher mortality; a paucity of randomized controlled evidence exists for whether correction of hypophosphatemia improves the outcome in critically ill patients.

**Conclusions:**

Additional studies addressing the current approach to hypophosphatemia in critically ill patients are required. Studies should focus on the association between hypophosphatemia and morbidity and/or mortality, as well as the effect of correction of this electrolyte disorder.

## Introduction

Electrolyte disorders frequently develop in critically ill patients during course of stay in the intensive care unit (ICU). Therefore, ICU patients are routinely monitored for electrolyte disorders, and it is common practice to correct them. Hypophosphatemia is one of those frequently encountered electrolyte disorders, for which many causative factors are present in critically ill patients. It is uncertain when and how to correct hypophosphatemia, and whether correction affects outcome in critically ill patients.

We searched the literature on hypophosphatemia in ICU patients to identify the incidence, symptoms, and treatment of hypophosphatemia. We searched for answers to the following questions: (a) whether correction of hypophosphatemia is associated with improved outcome; and (b) whether a certain treatment strategy is superior.

## Materials and methods

The Medline database was searched to identify articles from 1969 to 2010 containing the Medical Subjects Heading (MeSH) term "hypophosphatemia." We included clinical studies and experimental trials, as well as case reports. Results were limited to articles in the English language and to articles on humans. This search yielded 1,413 articles. The Cochrane Library was also searched for current trials on hypophosphatemia, which yielded no results. All articles were screened for relevance to critically ill patients; these articles were studied in detail. Notably, articles on chronic hypophosphatemia (for example, hereditary hypophosphatemic syndromes) were excluded.

## Results

### Phosphate metabolism and causes of hypophosphatemia in critically ill patients

**Figure 1:**
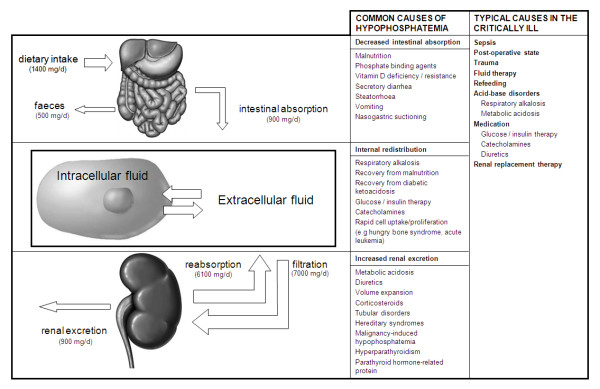
**Phosphate metabolism and causes of hypophosphatemia**.

Phosphorus is an essential element for all living cells, with different functions (Table 1) [1]. The phosphate balance is a complex interplay between phosphate uptake and phosphate excretion (Figure 1). Normal values of the total serum phosphate level are 0.80 to 1.45 mmol/L (2.5 to 4.5 mg/dl).

**Table 1:** Functions of phosphate

Form	Function
Hydroxyapatite	Bone structure
Phospholipids	Structure of cell membranes
Adenosine triphosphate (ATP) and creatine phosphate	Energy storage and metabolism
Nucleic acids and nucleoproteins	Genetic translation
Phosphorylation of proteins	Key regulatory mechanism; activation of enzymes, cell-signaling cascade
2,3-Diphosphoglycerate	Modulates oxygen release by hemoglobin
Inorganic phosphate	Acid-base buffer

Hypophosphatemia can be caused by three different mechanisms [1,2]: decreased intestinal absorption, increased renal excretion, or internal redistribution of inorganic phosphate (Figure 1). In most patients with severe hypophosphatemia, both depletion of total body phosphorus stores and redistribution of phosphate to the intracellular space are found. Decreased intestinal absorption of phosphate rarely causes hypophosphatemia, as a low-phosphate diet increases renal reabsorption and enhances intestinal uptake of phosphate. Still, malnutrition, diarrhea, and nasogastric suction are common features in critically ill patients.

Redistribution across the cell membrane is the most common cause of hypophosphatemia in ICU patients and can be caused by multiple clinical conditions [1,3]: respiratory alkalosis-induced increase of intracellular pH causes phosphate to enter the cell by stimulating glycolysis [4]; administration of glucose and insulin also stimulates carbohydrate metabolism, during which phosphate is transported into the cells along with glucose; high serum levels of catecholamines such as epinephrine and norepinephrine, whether endogenous or exogenous, cause a decrease in serum phosphate [5]; cellular uptake of phosphate is increased under certain specific conditions such as the hungry-bone syndrome, and diseases with rapid cell proliferation such as acute leukemia; renal excretion of phosphate is increased by metabolic acidosis, and by many drugs, including diuretics, glucocorticoids [6], aminoglycosides, antiretroviral drugs, and anticancer drugs.

Serum phosphate levels are inversely correlated to levels of the inflammatory cytokines interleukin-6 and tumor necrosis factor-α [7]. The exact mechanism is unclear: renal phosphate excretion is very low in patients receiving interleukin therapy for cancer [8], suggesting that high interleukin levels cause internal redistribution of phosphate; hypophosphatemia may be caused by increased phosphate utilization by immune cells. Hypophosphatemia can be found in patients with severe infections, such as sepsis. Especially patients with Gram-negative bacteremia may develop hypophosphatemia [9]. Hypophosphatemia correlates to severity of illness and can even be used as a prognostic parameter in sepsis patients [10]. Infection with *Legionella *species is particularly associated with hypophosphatemia [11].

Hypophosphatemia often develops in the postoperative phase [12-15]. Multiple causal factors may be present, such as respiratory alkalosis, administration of insulin, and the use of diuretics. This is particularly true for major surgery, such as cardiac surgery and abdominal aortic surgery. The role of cardiopulmonary bypass is not clear. After major hepatic surgery, hypophosphatemia is extremely frequent. Reported mechanisms involve both shifts of phosphate into hepatocytes [16] and renal phosphate wasting [17]. Hypophosphatemia is reported to be more frequently encountered in trauma patients [18]. Renal phosphate handling is altered in trauma patients, resulting in inadequately increased urinary phosphate excretion. Hypophosphatemia is even more frequent in burn-wound victims, where phosphate is lost through the skin [19,20]. In patients with head trauma, induction of polyuresis may be an aggravating factor [21].

In patients with malnutrition, a so-called refeeding syndrome may develop when they receive (par-)enteral feeding, a syndrome characterized by multiple metabolic abnormalities including depletion of total body phosphorus stores and redistribution of phosphate to the intracellular compartment, which may result in severe hypophosphatemia [22].

Hypothermia induces polyuresis and is associated with hypophosphatemia as well [23]. The use of (continuous) renal replacement therapy may lead to hypophosphatemia when low-phosphate replacement solution and dialysate are used. Patients who require high-flux dialysis for intoxications are especially at risk. Addition of potassium phosphate to dialysate and replacement fluids safely prevents the development of hypophosphatemia [24].

Finally, patients with diabetic ketoacidosis commonly present with hypophosphatemia due to increased urinary phosphate excretion. Phosphate levels generally decrease further during treatment because of intracellular shifting along with glucose and potassium [25].

### Epidemiology of hypophosphatemia

Table 2 summarizes the reported incidence and prevalence of hypophosphatemia in surgical and medical ICU patients [7,12,14,15,17-19,21,26-30]. Hypophosphatemia is usually categorized as moderate (serum phosphate level of 0.32 to 0.65 mmol/L (1 to 2 mg/dl) or severe (<0.32 mmol/L (<1 mg/dl)). In the general hospital population, the prevalence of moderate hypophosphatemia ranges between 2.2 and 3.1% [31,32], and the prevalence of severe hypophosphatemia is reported to be 0.2 to 0.4% [32-34]. One study reports that 45% of all hospital hypophosphatemia cases occur in the ICU population [35]. Hypophosphatemia has a higher incidence in certain patient groups, such as patients with diabetic ketoacidosis, sepsis, and postoperative patients. Hypophosphatemia is found in as many as 34% of patients after elective cardiac surgery [12]. An extremely high incidence of hypophosphatemia is reported after major hepatic surgery, where almost all patients develop hypophosphatemia in the first postoperative week [17,26]. In this group, serum phosphate levels decrease to a nadir within approximately 2 days and recover in the following days. This early nadir is also described after cardiac surgery and in patients with diabetic ketoacidosis and the refeeding syndrome. Trauma patients have a higher incidence of hypophosphatemia, especially patients with burn wounds [19] and head trauma [21]. Although the use of renal replacement therapy leads to hypophosphatemia, no epidemiologic reports were found.

**Table 2:** Prevalence and/or incidence of hypophosphatemia

Author [ref.]	Year	Population/disease	Number of patients	Definition of hypophosphatemia	Prevalence	Incidence
Surgical ICU patients						

Goldstein *et al. *[15]	1985	Thoracic surgery	34	<0.80 mmol/L	-	56%
		Cardiac surgery	40	<0.80 mmol/L	-	50%
Zazzo *et al. *[14]	1995	Surgical ICU	208	<0.80 mmol/L	-	28.8%
				≤0.50 mmol/L	-	17.3%
				≤0.20 mmol/L	-	2.4%
Buell *et al. *[26]	1998	Hepatic surgery	35	<0.80 mmol/L	-	67%
Cohen *et al. *[12]	2004	Cardiac surgery	566	<0.48 mmol/L	-	34.3%
Salem *et al. *[17]	2005	Hepatic surgery	20	<0.70 mmol/L	-	100%

Medical ICU patients						

Daily *et al. *[18]	1990	Trauma patients	12	<0.80 mmol/L	-	75%
				<0.50 mmol/L	-	56%
Kruse *et al. *[27]	1992	General ICU patients	418	<0.80 mmol/L	-	28%
Marik *et al. *[28]	1996	Refeeding after >48 h starvation	62	<0.65 mmol/L	-	34%
				<0.32 mmol/L	-	6%
Berger *et al. *[19]	1997	Burn injuries	16	<0.80 mmol/L	-	100%
				<0.30 mmol/L	-	50%
Barak *et al. *[7]	1998	Sepsis	99	<0.80 mmol/L	80%	-
		Infection without sepsis	32	<0.80 mmol/L	65%	-
		Sepsis, negative blood culture	37	<0.80 mmol/L	80%	-
		Sepsis, postive blood culture	30	<0.80 mmol/L	80%	-
Polderman *et al. *[21]	2000	Head trauma	18	<0.60 mmol/L	61%	-
Milionis *et al. *[29]	2002	Severe heart failure	86	<0.77 mmol/L	13%	-
Dominguez-Roldan *et al. *[30]	2005	Brain-dead patients	50	<0.80 mmol/L	-	72%

Correction of hypophosphatemia, when encountered, is not reported in epidemiologic studies and the spontaneous course of serum phosphate levels without treatment is generally not addressed.

### Symptoms of hypophosphatemia

Serum phosphate levels do not accurately reflect total body phosphorus stores; hence the degree of hypophosphatemia does not always correlate to the presence of symptoms. Although most patients with hypophosphatemia do not develop symptoms, fatal complications have been described. A common mechanism in hypophosphatemia-caused complications is impaired energy metabolism, leading to cellular dysfunction in multiple organ systems. Symptoms are summarized in Table 3.

**Table 3:** Symptoms of hypophosphatemia

**Respiratory**

Respiratory muscle dysfunction
Acute respiratory failure
Failure to wean from mechanical ventilation
Decreased peripheral oxygen delivery

**Cardiovascular**

Decreased myocardial contractility
Acute heart failure
Increased inotropic requirement
Arrhythmia
Ventricular tachycardia
Supraventricular tachycardia
Premature beats

**Hematologic**

Hemolysis
Leukocyte dysfunction

**Endocrine**

Insulin resistance

**Neuromuscular**

Skeletal muscle weakness
Rhabdomyolysis
Polyneuropathy
Altered mental status
Seizures
Encephalopathy
Central pontine myelinolysis

#### Respiratory effects

Hypophosphatemia is associated with respiratory muscle dysfunction, potentially resulting in (acute) respiratory failure and weaning problems [36-38]. The mechanism is considered to be decreased availability of phosphate-containing energy sources. Depletion of 2,3- diphosphoglycerate (2,3-DPG) shifts the oxygen dissociation curve to the left, decreasing oxygen delivery to peripheral tissue [39,40]. This might be especially relevant in patients with chronic pulmonary disease, as these patients may have higher 2,3-DPG levels to compensate for hypoxemia. In addition, hypophosphatemia has also been associated with decreased tissue oxygenation after correction for 2,3-DPG levels [41].

#### Cardiovascular effects

Hypophosphatemia can lead to myocardial dysfunction and arrhythmias. Phosphate depletion causes impaired energy metabolism in the myocardium, leading to decreased contractility [42,43]. Severe acute heart failure has been described in several case reports in the presence of severe hypophosphatemia. Hypophosphatemia after cardiac surgery was associated with higher requirements of inotropic support [12]. Correction of hypophosphatemia is associated with improved cardiac output [14]. Hypophosphatemia is a significant predictor of ventricular tachycardia after myocardial infarction [44] and a correlation with arrhythmias has been suggested in septic patients [45]. During correction of hypophosphatemia, phosphate may precipitate with calcium and cause hypocalcemia. It is important to keep in mind that hypocalcemia can negatively influence cardiac function as well.

#### Other effects

Hypophosphatemia can cause hematologic dysfunction [46-48], insulin resistance [49], and a number of neuromuscular symptoms (Table 3). Of the latter, rhabdomyolysis [50,51] and central pontine myelinolysis [52,53] are most severe. Besides hypophosphatemia, critically ill patients frequently have multiple factors putting them at risk for neurologic alterations, and causality is not well documented.

#### Hypophosphatemia and mortality

Multiple studies show an association between hypophosphatemia and increased mortality [10,12,14,35,54-59]. Severe hypophosphatemia has been reported to predict up to eightfold increased mortality rate in sepsis patients [10]. However, hypophosphatemia has not been associated with increased mortality after cardiac surgery [12] and in diabetic ketoacidosis [54]. It remains unclear whether hypophosphatemia actually contributes to mortality, or merely is a marker for severity of illness. Whether correction of hypophosphatemia reduces mortality is currently unknown.

### Correction of hypophosphatemia

With the high prevalence of hypophosphatemia in critically ill patients, as well as their susceptibility to life-threatening symptoms, frequent laboratory monitoring is recommended, especially in previously mentioned high-risk groups. It is generally recommended to correct hypophosphatemia in hypophosphatemic patients with associated symptoms [2,60]. However, no randomized controlled evidence indicates whether correction of hypophosphatemia in apparently asymptomatic patients leads to improved outcome. Taking this into account, the indication for -- and recommended frequency of -- laboratory monitoring and treatment remains debatable.

Correction of hypophosphatemia is possible via oral or intravenous routes. Intravenous administration of phosphate is not without complications, though. Phosphate may precipitate with calcium. Large intravenous doses of phosphate may result in hyperphosphatemia, hypomagnesemia, hypocalcemia, and hypotension. It is therefore necessary to know when intravenous therapy is indicated, and how much and how fast phosphate should be supplied. Intravenous therapy is generally recommended in symptomatic hypophosphatemia and phosphate levels <0.32 mmol/L. Multiple studies have evaluated the efficacy and safety of intravenous phosphate repletion regimens (Table 4) [61-67]. These studies generally agree that aggressive phosphate supplementation is safe with phosphate doses up to 45 mmol with infusion rates up to 20 mmol per hour. Hyperkalemia is prevented by using sodium phosphate instead of potassium phosphate in patients with potassium levels >4 mmol/L.

**Table 4:** Intravenous treatment of hypophosphatemia

Author [ref.]	Year	Serum phosphate (mmol/L)	Dose	Speed	Efficacy	Complications/safety
Brown *et al. *[61]	2006	0.73-0.96	0.32 mmol/kg	7.5 mmol/h	No significant increase in iP	Considered safe
		0.51-0.72	0.64 mmol/kg	7.5 mmol/h	iP normalized in 59%	Considered safe
		<0.50	1 mmol/kg	7.5 mmol/h	iP normalized in 60%	Considered safe
Taylor *et al. *[62]	2004	0.55-0.70	0.2 mmol/kg	33 μmol/kg/h	iP normalized in 76% (all patients)	Considered safe
		0.32-0.55	0.4 mmol/kg	67 μmol/kg/h		Considered safe
		<0.32	0.6 mmol/kg	100 μmol/kg/h		Considered safe
Charron *et al. *[63]	2003	0.40-0.65	30 mmol	15 mmol/h	Equally effective	Mild hyperphosphatemia and mild hyperkalemia
			30 mmol	7.5 mmol/h		
		<0.40	45 mmol	15 mmol/h	Equally effective	
			45 mmol	7.5 mmol/h		
Perreault *et al. *[64]	1997	0.40-0.80	15 mmol	5 mmol/h	iP normalized in 81.5%	Considered safe
		<0.40	30 mmol	10 mmol/h	iP normalized in 30%	Considered safe
Rosen *et al. *[65]	1995	0.50-0.65	15 mmol	7.5 mmol/h	iP normalized in 100%	Considered safe
Bollaert *et al. *[66]	1995	<0.65	20 mmol	20 mmol/h	iP normalized in 80%	Considered safe Mild hypocalcemia
Kruse *et al. *[67]	1992	<0.80	20-40 mmol	20 mmol/h	mean iP rose from 0.65 to 1.0 mmol/L	considered safe Mild hypocalcemia

iP, serum inorganic phosphate.

Moderate hypophosphatemia can be treated with oral supplementation of phosphate. One should keep in mind that active vitamin D is required for intestinal absorption of phosphate. Typical oral supplementation amounts are three times the normal daily intake, with advised amounts of 2.5 to 3.5 g (80 to 110 mmol) per day, divided over two to three doses. Patients who receive feeding after a period of starvation are often phosphate depleted, so additional phosphate should be added to nutritional preparations. An additional preventive strategy is to build up the caloric intake slowly [22]. The total required amount of phosphate cannot be predicted by serum phosphate levels, as phosphate shifts between multiple body compartments.

Dipyridamole can decrease urinary phosphate loss [68]. Further research is needed to establish further the role of this drug in the treatment of hypophosphatemia in critically ill patients.

## Discussion

Critically ill patients have a high prevalence of hypophosphatemia because of the presence of multiple causal factors. Hypophosphatemia may lead to a multitude of symptoms, but most often remains asymptomatic. Hypophosphatemia, however, is associated with increased mortality in important patient subgroups. It is important to investigate whether hypophosphatemia causes higher mortality in itself, or rather is associated with a higher severity of illness.

Because of the current paucity of evidence, serum phosphate levels are not routinely measured in all critically ill patients. The spontaneous course of serum phosphate is not well documented in the literature, so it is insufficiently clear whether an initially low phosphate level after surgery will return to normal spontaneously. Although multiple studies have evaluated the efficacy and safety of phosphate repletion regimens, the effect on mortality and morbidity is not well reported. Currently, no widely agreed-on guidelines exist concerning the approach to hypophosphatemia in critically ill patients, because evidence is lacking on when and how to treat hypophosphatemia.

A reasonable approach awaiting randomized controlled trial evidence would be to reserve intravenous correction of hypophosphatemia for patients with associated symptoms or phosphate levels <0.32 mmol/L. Doses of up to 40 mmol of sodium phosphate, administered at a speed of up to 20 mmol per hour, are proven to be safe. Additional studies are required, addressing the current approach to hypophosphatemia in critically ill patients, as well as the association of hypophosphatemia with morbidity and mortality, and the effect of treatment. Evidence-based guidelines are needed to guide critical care physicians in the diagnosis and treatment of hypophosphatemia.

## Conclusions

Additional studies are required to address the current approach to hypophosphatemia in critically ill patients, as well as the association of hypophosphatemia with morbidity and mortality, and the effect of the correction of this electrolyte disorder.

## Key messages

• Critically ill patients have a high prevalence of hypophosphatemia because of the presence of multiple causal factors.

• Hypophosphatemia may lead to a multitude of symptoms, including cardiac and respiratory failure, and is associated with higher mortality.

• Although multiple studies confirm the efficacy and safety of intravenous phosphate administration, it is unknown which treatment strategy is superior.

• Nevertheless, correction of hypophosphatemia has not been shown to improve outcome.

## Abbreviations

2,3-DPG: 2,3-diphosphoglycerate; ATP: adenosine triphosphate; ICU: intensive care unit.

## Competing interests

The authors declare that they have no competing interests.

## Authors' contributions

DAG searched the literature, interpreted the results, and drafted the manuscript. DAG, AJB, MAK, ANR, PES, and MJS participated in drafting and reviewing the manuscript. All authors approved the final version of the manuscript.
